# Dispersible formulation of artemether/lumefantrine: specifically developed for infants and young children

**DOI:** 10.1186/1475-2875-8-S1-S7

**Published:** 2009-10-12

**Authors:** Salim Abdulla, Issaka Sagara

**Affiliations:** 1 Ifakara Health Institute Dar-es-Salaam, United Republic of Tanzania; 2 Malaria Research and Training Center, Faculty of Medicine, Pharmacy and Odonto-Stomatology, University of Bamako, Bamako, Mali

## Abstract

Infants and children under five years of age are the most vulnerable to malaria with over 1,700 deaths per day from malaria in this group. However, until recently, there were no WHO-endorsed paediatric anti-malarial formulations available.

Artemisinin-based combination therapy is the current standard of care for patients with uncomplicated falciparum malaria in Africa. Artemether/lumefantrine (AL) meets WHO pre-qualification criteria for efficacy, safety and quality. Coartem^®^, a fixed dose combination of artemether and lumefantrine, has consistently achieved cure rates of >95% in clinical trials. However, AL tablets are inconvenient for caregivers to administer as they need to be crushed and mixed with water or food for infants and young children. Further, in common with other anti-malarials, they have a bitter taste, which may result in children spitting the medicine out and not receiving the full therapeutic dose. There was a clear unmet medical need for a formulation of AL specifically designed for children.

Ahead of a call from WHO for child-friendly medicines, Novartis, working in partnership with Medicines for Malaria Venture (MMV), started the development of a new formulation of AL for infants and young children: Coartem^® ^Dispersible. The excellent efficacy, safety and tolerability already demonstrated by AL tablets were confirmed with dispersible AL in a large trial comparing the crushed tablets with dispersible tablets in 899 African children with falciparum malaria. In the evaluable population, 28-day PCR-corrected cure rates of >96% were achieved. Further, its sweet taste means that it is palatable for children, and the dispersible formulation makes it easier for caregivers to administer than bitter crushed tablets. Easing administration may foster compliance, hence improving therapeutic outcomes in infants and young children and helping to preserve the efficacy of ACT.

## Background

Launched on 6 December 2007, *'Make medicines child size*' is a five-year global campaign spearheaded by the World Health Organization (WHO) [1]. The aim is to raise awareness and accelerate action to address the need for improved availability and access to safe and effective child-specific medicines for all children under the age of 15. To achieve this goal, more research is needed, more medicines need to be developed, and improved access measures are essential. At present, many medicines are not developed specifically for children or with the specific needs of children taken into account, and are often not available in suitable dosage forms.

The *'Make medicines child size*' campaign is aiming to change that reality. Both EU and US legislation encourage the development of paediatric formulations and there is now a shift from protecting children from clinical trials to protecting them through clinical trials. As children tend to metabolize medicines differently to adults, they need to be given a tailored dosage in a suitable, stable format that is palatable and easy to administer. Small children have difficulty swallowing whole tablets, but other formulations, such as dispersible tablets or syrups, are much easier to take.

According to WHO, over nine million children under five years old die each year, with over 1,700 malaria-related deaths every day in this group [2]. Access to prompt and effective treatment of malaria is the main challenge in Africa, although dose accuracy and adherence to the treatment schedule are equally important to ensure an adequate therapeutic response [3].

Despite the fact that infants and children bear the greatest burden of malaria, there has been, until recently, no anti-malarial agent specifically formulated for this vulnerable group. Research into the development of alternative formulations for children has been limited, and although specific paediatric formulations such as syrups and suspensions exist (e.g. artemether/lumefantrine suspension [4]), these still may involve reconstitution or volume measurement in the field. Therefore, there is still a need for a formulation that facilitates easy and accurate dosing.

## Addressing an unmet need

To date, artemisinin derivatives are the only class of anti-malarial agents to which parasite resistance has not been reported in Africa. WHO guidelines specifically recommend the use of artemisinin-based combination therapy (ACT) for the treatment of uncomplicated falciparum malaria [5]. Artemether/lumefantrine (AL) is an ACT that meets WHO prequalification criteria for efficacy, safety and quality [6] and AL (Coartem^®^) has been recently approved by the USA FDA [7].

Coartem^®^, a fixed dose combination of artemether and lumefantrine, has consistently achieved cure rates of >95% in clinical trials of children with malaria and was shown to be safe and well tolerated [8,9].

AL tablets are available in paediatric doses; however, they must be crushed for infants and small children as whole tablets may present a choking hazard. The crushed tablets, in common with many anti-malarials, have a bitter taste that may cause children to spit them out, and may result in a sub-therapeutic dose being taken. Further, crushing of the tablets by caregivers at home may result in loss of active ingredients and thus under dosing. Together, these factors could result in an opportunity for parasite resistance to develop.

### A public-private partnership

Recognizing that few medicines have been successfully created to address the needs of children with malaria, Novartis and Medicines for Malaria Venture (MMV) formed a public-private partnership with the aim of developing a paediatric formulation of AL. MMV is a global, non-profit organization created to discover, develop and deliver safe, effective and affordable anti-malarials for vulnerable populations, whilst Novartis is a global pharmaceutical company with a wealth of experience in drug development and a team of investigators in endemic countries. Pooling their resources meant that the partners were ideally placed to develop a formulation of AL specifically designed for infants and young children.

## Challenges in developing a paediatric formulation

The biggest challenge in developing a new formulation is to ensure that it is acceptable to all stakeholders. For patients, it must be palatable and easy to take. For caregivers, accessibility and ease of administration to a sick child is a prime concern. For regulatory authorities, there must be sufficient data to support the use of the new formulation in the target population in terms of quality, efficacy and safety. Purchasers are most concerned about cost and availability.

The choice of which paediatric formulation to develop was carefully considered. Both syrups and powder for reconstitution, which are used for existing paediatric drug formulations, have several disadvantages: they are bulky to supply and store, and once opened/reconstituted, the stability and hygiene of the formulations can no longer be guaranteed. Accurate dosing of syrups and powder for reconstitution, typically delivered in a multi-dose format, may be difficult as it requires accurate volume measurement in the field.

The development of a dispersible tablet of AL allowed an accurate dose of the active ingredient to be contained in a tablet form, and for the dosing schedule to match that of existing AL tablets. The dispersible tablets are simpler for caregivers to prepare and administer than crushed bitter tablets and easier for sick children and infants to take [10]. They require only a small amount of water for dispersion [10]. The availability of clean, safe water for dispersion of the tablet is not considered to be an issue, as water is needed to facilitate swallowing of any tablet, whether a crushed AL tablet or another medication. Finally, the existing colour-coded packaging with clear step-by-step instructions for use could easily be adapted for the packaging of a dispersible AL formulation, building on the education and understanding that has already been achieved.

### Palatability

Palatability is a contributing factor to compliance in children's medicines [11]. The success of dispersible AL in terms of acceptability to children was dependent on a flavour that masks the bitter taste.

A randomized, single-centre cross-over study (Study COA566B2101, Novartis Pharma AG. Reference Investigators - Abdulla *et al. *Personal Communication) was carried out with healthy African schoolchildren to evaluate the palatability of three flavours of AL for oral suspension. Strawberry, orange and cherry flavours were tested, and overall, cherry was found to be the preferred flavour. Even though this fruit is not native to Africa it is often found in soft drinks and other medications such as antibiotic syrups.

## Dispersible artemether/lumefantrine development programme

Once a paediatric formulation of AL had been developed it was subjected to a rigorous clinical development programme consisting of several key steps: testing the formulation for stability in hot and humid conditions, comparing the bioavailability with current marketed AL tablets in children with malaria, and demonstrating clinical non-inferiority (efficacy and safety) to the marketed AL tablets in children with malaria. The culmination of the programme was filing for approval with Swissmedic (ICH stringent regulatory authority) and launch across Africa following registration in endemic countries.

## Bioavailability of dispersible artemether/lumefantrine

Before embarking on a large efficacy and safety clinical study in children, the pharmacokinetic (PK) performance of the dispersible tablet was investigated and compared to regular AL tablets. The study objective was to assess bio-availability of artemether, dihydroartemisinin (DHA, the active metabolite of artemether) and lumefantrine (Study COA566B2104, Novartis Pharma AG. Reference Investigators - Abdulla *et al. *Personal Communication). In this randomized, open-label, single-dose crossover study, 48 healthy European adult volunteers with a mean age of 33.1 ± 7.8 years (range 22-50 years) were recruited. Single doses of dispersible AL tablets or crushed regular AL tablets were administered after a FDA standard breakfast, with 4-week washout periods. Rich plasma sampling was performed for comprehensive PK evaluation of artemether, DHA and lumefantrine. Similar PK profiles were found with crushed and dispersible tablets in healthy adults for all three test compounds.

A PK comparison in children with malaria was undertaken as part of a randomized, multicentre, investigator-blinded study in African children [12]. Similar PK profiles were reported for artemether, DHA and lumefantrine in the dispersible and the crushed tablet formulations.

## Efficacy of dispersible artemether/lumefantrine

AL tablets have achieved cure rates of >95% in children with falciparum malaria [8,9]. The efficacy of the new dispersible AL tablets was compared with that of crushed AL tablets in a study of 899 African children with uncomplicated falciparum malaria [12]. In summary, this was a randomized, multi-centre, two-arm, investigator blinded trial in five African countries (Benin, Mali, Kenya, Mozambique and Tanzania/Zanzibar).

**Figure 1:**
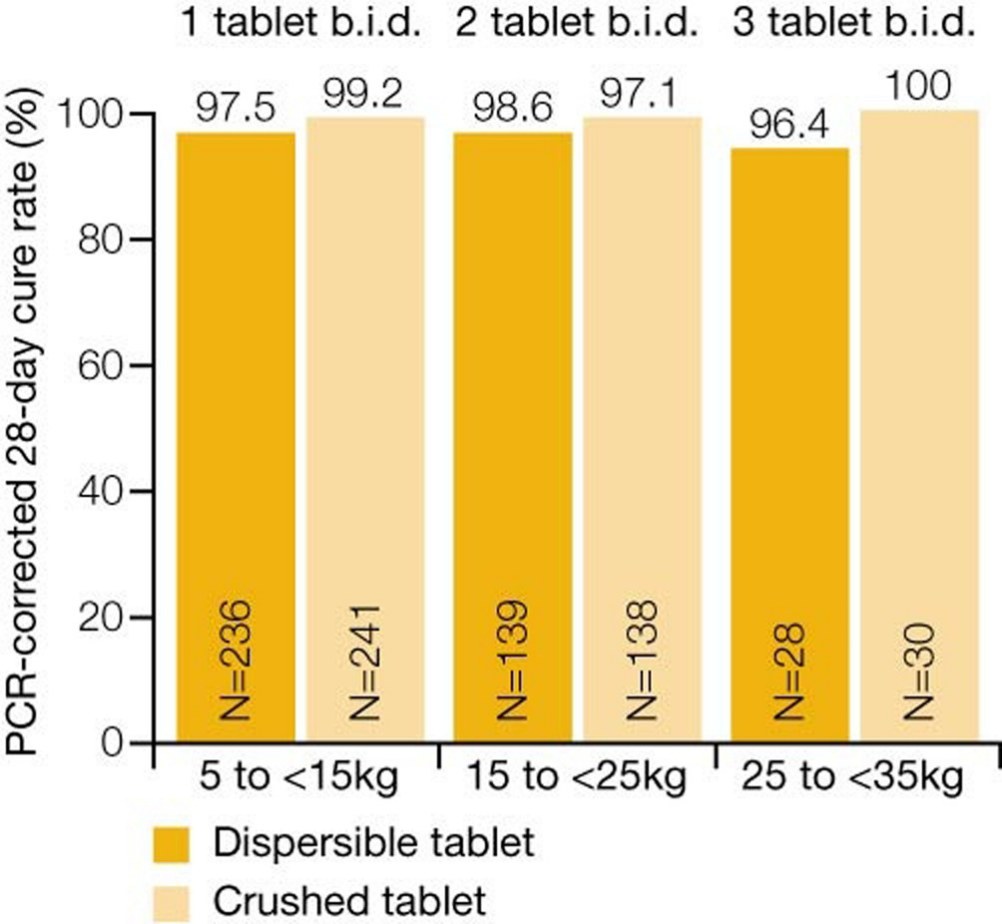
**Similar efficacy across body weight groups with crushed AL tablet and dispersible AL tablet (PCR-corrected cure rate in the modified intent-to-treat population)**. Adapted from Abdulla *et al *[12].

**Figure 2:**
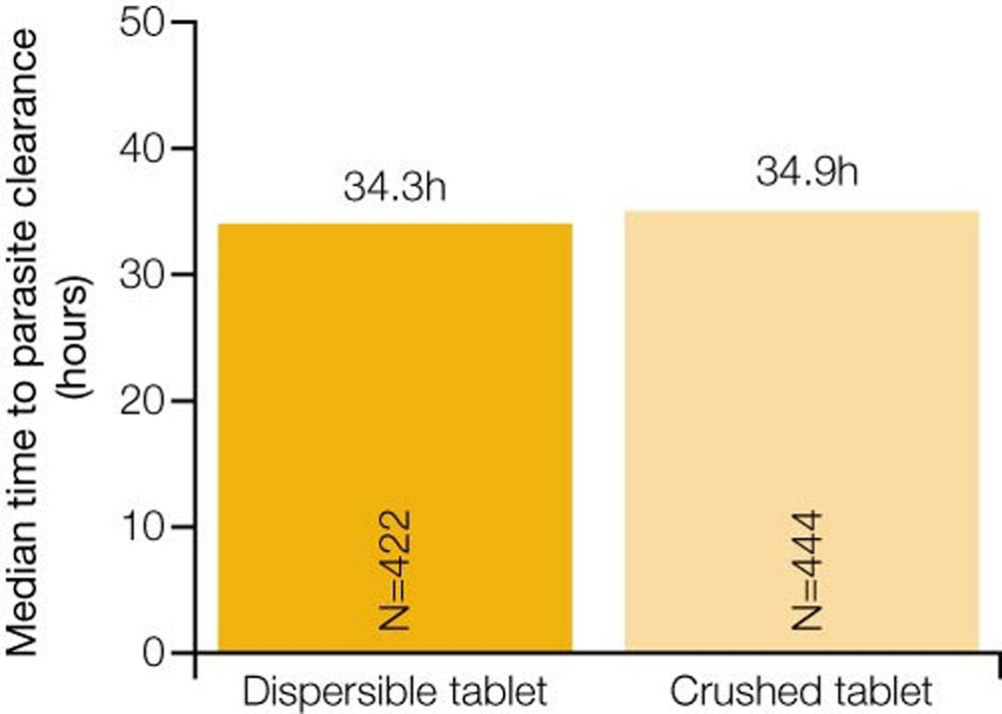
**Similar median time to parasite clearance with crushed AL tablets and dispersible AL tablets (among patients with fever at baseline)**. Adapted from Abdulla *et al *[12].

**Figure 3:**
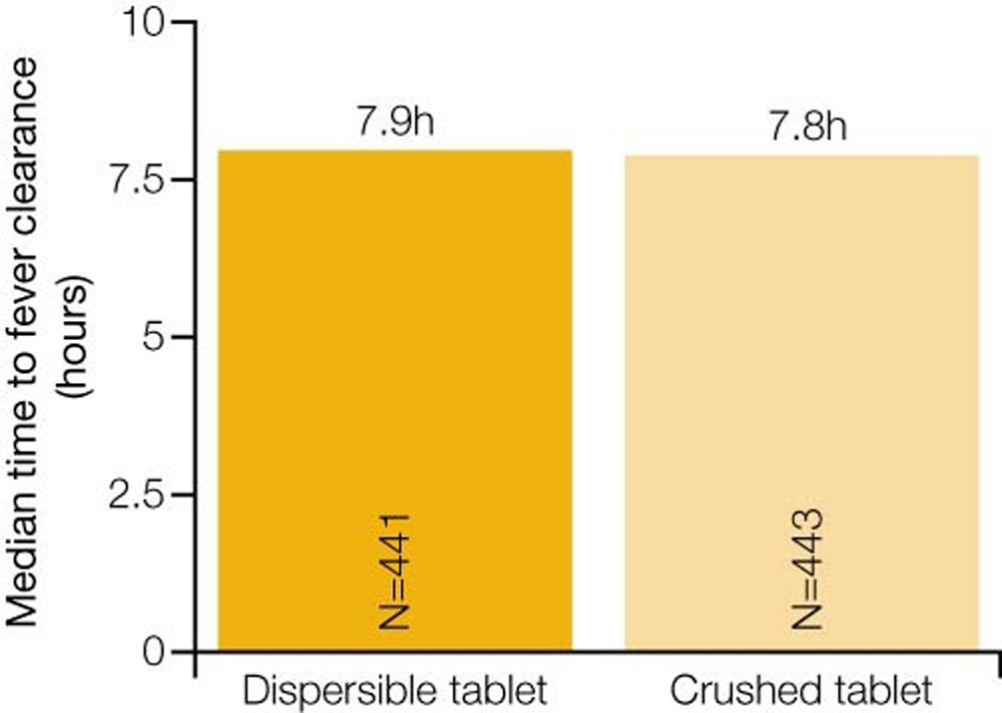
**Similar median time to fever clearance is with crushed AL tablets and dispersible AL tablets**. Adapted from Abdulla *et al *[12].

In the evaluable population, the 28-day PCR-corrected cure rate was 98.5% for the group receiving crushed AL tablets and 97.8% for the group receiving dispersible AL tablets [12]. The PCR-adjusted cure rates for the two groups were also comparable at day 14 and day 42 [12]. Notably, the cure rates were similar across three different body weight categories/dosing regimen groups (Figure 1) [12]. In addition, there were no significant differences between those receiving crushed tablets and those receiving dispersible tablets with regard to median time to parasite clearance and median time to fever clearance (Figures 2 and 3) [12].

Only a small proportion of patients had gametocytes after day 8 (crushed tablet 1.2%, dispersible tablet 0.5%) [12]. Gametocyte clearance could not be assessed as too few patients had gametocytes at baseline. However, the low prevalence of gametocytes post-treatment with AL observed in this large study population suggests that this treatment results in low gametocyte carriage and so may reduce malaria transmission in highly endemic areas.

## Safety

AL tablets have demonstrated an excellent safety profile in children with falciparum malaria [8,9]. The safety and tolerability of dispersible AL tablets was examined in the same randomized study of 899 African children (5-≤ 35 kg body weight) and showed a similar pattern and incidence of adverse events as crushed AL tablets [12].

Tolerability was good with both crushed and dispersible tablets, with no difference in the pattern and overall incidence of adverse events (AEs) across both formulations. No new or unexpected AEs were seen in either treatment group. Most commonly reported AEs were related to malaria (e.g. pyrexia), with only 12.4% (56) and 9.4% (42) of patients with an AE suspected to be related to the crushed tablets or the dispersible tablets respectively. The most frequent drug-related AE was vomiting, but only a fraction of patients needed rescue medication due to vomiting of study medication (2.4% in the crushed tablet group; 1.3% in the dispersible tablet group). Vomiting was more frequently reported in the lowest body weight category. Other drug-related AEs occurred in less than 0.9% of patients in either group, and no clinically relevant changes in vital signs or laboratory evaluations were observed.

There were three deaths during the study. In the group taking dispersible tablets, one patient died of haemorrhage following scarification by a traditional healer, and one from an unspecified infection accompanied by severe dehydration. One patient in the crushed tablet group died of severe *P. falciparum *malaria (new infection).

## Dispersible artemether/lumefantrine packaging

**Figure 4:**
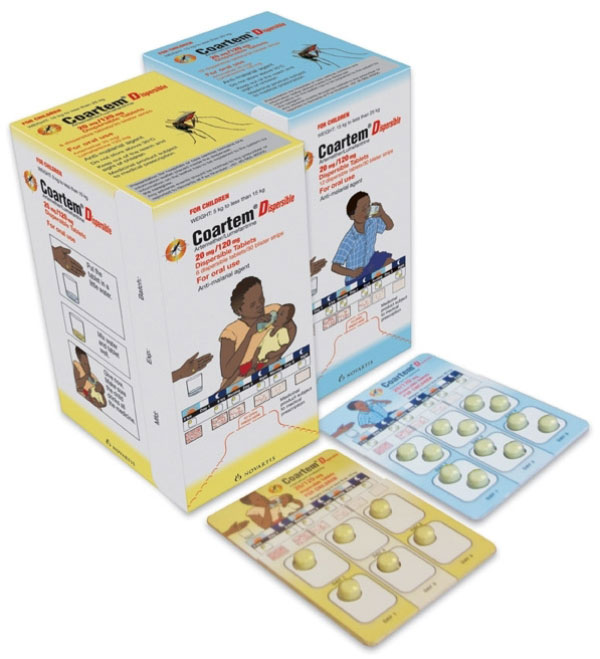
**Coartem^® ^Dispersible packaging**.

**Figure 5:**
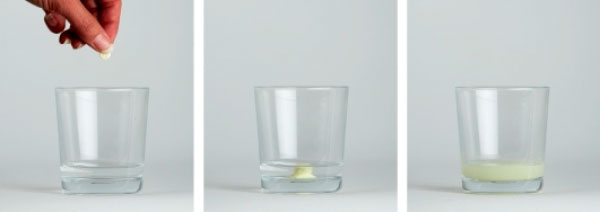
**Step-by-step instructions on how to make up the dispersible AL tablets for easy administration to children**. The tablet disperses in a small amount of water to produce a sweet-tasting solution.

Packaging for dispersible AL was created to further build on the legacy of the packaging that already exists for AL tablets. The packaging was designed based on independent field testing with rural African health workers, patients and community members, including mothers. As literacy levels vary, the packaging was made as graphical as possible (Figure 4). There are clear, three-step instructions on how to make up the dispersible AL and give the dispersed tablet(s) to the child (Figure 5). Key features of the packaging are diagrams that facilitate understanding of the dosing regimen and the need to complete the full three-day course. For example, the rationale for compliance with the three-day course of tablets is shown visually by the number of parasites decreasing as the course of medicine is completed. It clearly shows that patients must complete the full course to be cured.

The packaging serves as an educational tool that the health worker can use to teach the caregiver how and when to administer the dispersible AL and serves as a visual aid for the caregiver once at home. Ultimately, the packaging supports best practice in both local health service provision and patient use of dispersible AL. Further, it is easy to store and hygienic, with each dispersible tablet enclosed in a separate foil blister.

## Administration of dispersible artemether/lumefantrine and approval status

Dispersible AL tablets rapidly disperse (<1 min) in water to form a sweet-tasting medicine [10]. They require only a small amount of water to disperse in a spoon or a beaker and have no need for any special equipment [10]. They can be administered with food or milk [10]. Dispersible tablets are simpler for caregivers to prepare and administer than bitter crushed tablets [10] and will hopefully prove easier for infants and children to take.

Dispersible AL was approved by Swissmedic in December 2008 and received WHO prequalification in February 2009. It has been approved in 24 African countries to date.

## Conclusion

Dispersible tablets are expected to contribute to ease of administration of anti-malarial medication in those most vulnerable to malaria, i.e. infants and children. Further studies will be needed to establish the impact of the dispersible tablets on compliance, but it is hoped that the simplicity of administration will contribute greatly to adherence to the full three-day course of tablets, improving malaria morbidity and mortality in infants and young children and minimizing the opportunity for emergence of resistance. Novartis and MMV continue to work together to ensure the rapid delivery and adoption of dispersible AL to achieve optimal and affordable treatment of malaria that is tailored to the needs of children and their caregivers. Other partners such as African governments are striving to make sure that the dispersible tablets are reaching those people who need it the most, as well as organising training courses for healthcare workers to facilitate the switch from regular AL tablets to the dispersible formulation. This transition to the new formulation is not thought to present any significant challenges as the dosing pattern is the same as regular AL tablets. Procurement is underway; the dispersible tablets have already been delivered to countries such as Zambia and Mali, with more countries to follow imminently.

## Competing interests

The authors would like to acknowledge that Novartis Pharma AG sponsored this supplement. However, none of the authors works for, or represents in any way, Novartis Pharma AG.

## Authors' contributions

All authors met International Committee of Medical Journal Editors criteria for authorship.
